# Cross-Sectional Area of the Tibial Nerve in Diabetic Peripheral Neuropathy Patients: A Systematic Review and Meta-Analysis of Ultrasonography Studies

**DOI:** 10.3390/medicina58121696

**Published:** 2022-11-22

**Authors:** Thanyaporn Senarai, Thongchai Pratipanawatr, Laphatrada Yurasakpong, Nutmethee Kruepunga, Jarukitt Limwachiranon, Phetcharat Phanthong, Krai Meemon, Kaissar Yammine, Athikhun Suwannakhan

**Affiliations:** 1 Electron Microscopy Unit, Department of Anatomy, Faculty of Medicine, Khon Kaen University, Khon Kaen 40002, Thailand; thansen@kku.ac.th; 2 Department of Internal Medicine, Faculty of Medicine, Khon Kaen University, Khon Kaen 40002, Thailand; thongcha@kku.ac.th; 3 Princess Srisavangavadhana College of Medicine, Chulabhorn Royal Academy, Bangkok 10210, Thailand; laphatrada.yur@cra.ac.th; 4 Department of Anatomy, Faculty of Science, Mahidol University, Bangkok 10400, Thailand; nutmethee.kru@mahidol.edu (N.K.); phetcharat.pha@mahidol.edu (P.P.); krai.mee@mahidol.edu (K.M.); 5 In Silico and Clinical Anatomy Research Group (iSCAN), Bangkok 10400, Thailand; 6 Department of Microbiology, School of Medicine, Zhejiang University, Hangzhou 310058, China; nampetch@zju.edu.cn; 7 The Children’s Hospital, Zhejiang University School of Medicine National Clinical Research Center for Child Health, Hangzhou 310058, China; 8 Department of Orthopedic and Trauma Surgery, Lebanese American University Medical Center—Rizk Hospital, Beirut 11-3288, Lebanon; cesaryam@gmail.com; 9 The Center for Evidence-Based Anatomy, Sports and Orthopedic Research, Lebanese American University, Byblos 11-3288, Lebanon

**Keywords:** peripheral neuropathy, ultrasound, diabetes, tibial nerve, cross-sectional area, meta-analysis

## Abstract

*Background:* There is a link between diabetic peripheral neuropathy (DPN) progression and the increase in the cross-sectional area (CSA) of the tibial nerve at the ankle. Nevertheless, no prior meta-analysis has been conducted to evaluate its usefulness for the diagnosis of DPN. *Methods:* We searched Google Scholar, Scopus, and PubMed for potential studies. Studies had to report tibial nerve CSA at the ankle and diabetes status (DM, DPN, or healthy) to be included. A random-effect meta-analysis was applied to calculate pooled tibial nerve CSA and mean differences across the groups. Subgroup and correlational analyses were conducted to study the potential covariates. *Results:* The analysis of 3295 subjects revealed that tibial nerve CSA was 13.39 mm^2^ (CI: 10.94–15.85) in DM patients and 15.12 mm^2^ (CI: 11.76–18.48) in DPN patients. The CSA was 1.93 mm^2^ (CI: 0.92–2.95, I^2^ = 98.69%, *p* < 0.01) larger than DPN-free diabetic patients. The diagnostic criteria of DPN and age were also identified as potential moderators of tibial nerve CSA. *Conclusions:* Although tibial nerve CSA at the ankle was significantly larger in the DPN patients, its clinical usefulness is limited by the overlap between groups and the inconsistency in the criteria used to diagnose DPN.

## 1. Introduction

Diabetic peripheral neuropathy (DPN) is the most common chronic complication in type 1 and type 2 diabetic patients, accounting for up to two-thirds of non-traumatic amputations [1]. This condition is defined as “the presence of symptoms and/or signs of peripheral nerve dysfunction in people with diabetes after exclusion of other causes”. Due to the increasing incidence of type 2 diabetes over the years [2], it is recommended that early detection and prevention of DPN should be prioritized at the primary care level [3]. Early intervention strategies help prevent foot ulcers, reduce the risk of amputation and injuries due to their insensate feet [4], and ameliorate the social and economic costs of diabetic foot disease [5]. Screening for symptoms and early signs of DPN is therefore critical in clinical practice to preserve the patient’s quality of life in the long term. While nerve conduction study remains the gold standard for the diagnosis of DPN [6], it is time consuming, requires a separate visit, and is not necessarily available in every setup. It is also associated with high costs, which is not recommended for screening. A simpler tool for the diagnosis of DPN is therefore needed, especially in the primary care setting, to enable early detection of DPN with minimum requirements.

The utilization of high-resolution ultrasound is a relatively more convenient diagnostic approach and has emerged as a promising tool for scrutinizing peripheral neuropathy. High-resolution ultrasound allows for the visualization of the affected nerve’s echotexture and size [7]. Previous studies [8,9,10,11,12,13,14,15,16] have examined the cross-sectional area of the tibial nerve and found that its CSA was relatively larger in patients with DPN than those of diabetic patients without DPN. The increase in tibial nerve CSA is consistent with the common signs of DPN along the tibial nerve distributions including the absence of ankle reflexes, the disturbance of vibration, pinprick, temperature, and light touch sensations. These reflex scores and sensory tests are part of the clinical examination when using the Toronto Clinical Neuropathy Scoring System (TCNS) [17], American Diabetes Association Criteria [18], and Michigan Neuropathy Screening Instrument (MNSI) [19].

Despite the potential use of tibial nerve CSA as a possible screening tool for DPN, no prior meta-analysis has been conducted to assess its feasibility in larger populations. Therefore, this meta-analysis was conducted to generate weighted pooled estimates of ultrasonic tibial nerve CSA at the ankle and the mean difference between DM and DPN patients.

## 2. Materials and Methods

This meta-analysis was performed in accordance with the PRISMA 2020 statement [20], the Checklist for Anatomical Reviews and Meta-analysis (CARMA) [21], and the Critical Appraisal Tool for Anatomical Meta-analysis (CATAM) [22]. The protocol employed in this meta-analysis was registered on PROSPERO (CRD42020218941).

### 2.1. Literature Search and Study Selection

**Figure 1:**
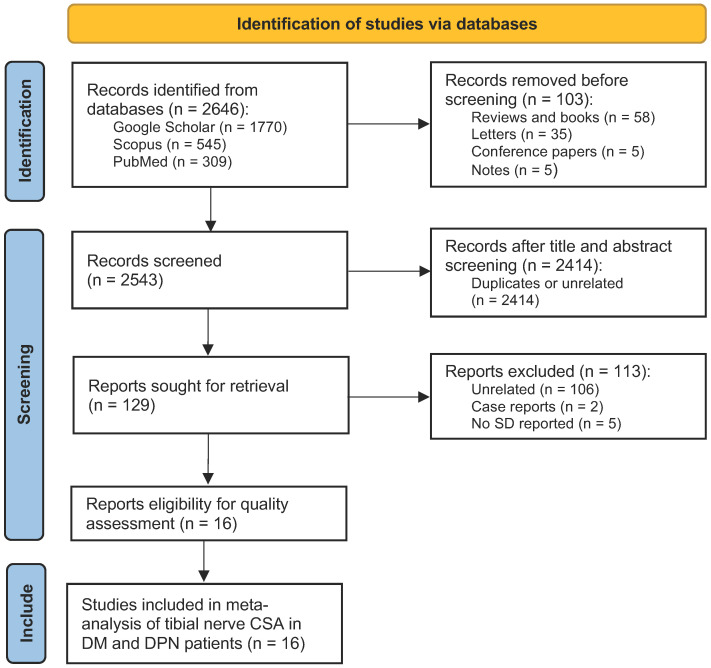
PRISMA flowchart summarizing the protocol of the present meta-analysis.

A systematic literature search was conducted as summarized in Figure 1. Searches were independently performed thorough Google Scholar, Scopus, and PubMed. For Google Scholar, the following keywords were used: “tibial nerve” AND (“ultrasound” OR ‘’sonography’’) AND “cross-sectional area”. For Scopus and PubMed, the following keywords were used: “tibial nerve” AND (“ultrasound” OR ‘’sonography” OR “cross-sectional area”). Reviews, letters books, notes, and conference papers were filtered out.

Study selection was performed independently with cross verification by two assessors. Studies were subject to further investigation if all of the following criteria were met: (1) tibial nerve CSA was reported; (2) tibial nerve CSA was measured by ultrasound; (3) the location of CSA measurement was reported; (4) the number of subjects were reported; (5) the included subjects had to be diagnosed with a specified type of diabetes with or without DPN; (6) the diagnostic criteria used to diagnose DPN was reported. Only studies that measured the tibial nerve CSA at the ankle (1–7 cm proximal to the medial malleolus) were included. Studies were subject to exclusion if (1) other methods rather than ultrasound were used to measure tibial nerve CSA or (2) the results were poorly or not clearly reported such as the lack of a standard deviation.

### 2.2. Quality Assessment

The methodological quality of the potential studies was further evaluated by the Revised-Quality Assessment of Diagnostic Accuracy Studies (QUADAS-2) tool [23]. Based on the original QUADAS, the QUADAS-2 tool has been designed to assess four aspects of methodological quality including: (1) patient selection; (2) details of the index test; (3) description of the reference standard; and (4) flow and timing of participant recruitment and outcome measurement. Each item was rated as “low risk”, “high risk”, or “unclear”. The scoring was performed by two of the authors. Quality assessment was performed only for 16 studies included in the meta-analysis of pooled tibial nerve CSA and the mean difference among DPN and DM patients. Emphasis was placed on the study design and how DPN or DM was diagnosed. The disagreement of quality assessment between the two authors, if any, was resolved by discussion until a consensus was reached.

### 2.3. Data Analysis and Data Synthesis

A meta-analysis was conducted to study the tibial nerve CSA and mean differences in DM patients, DPN patients, and healthy controls. The data that were extracted included tibial nerve CSA, standard deviations, number of subjects, geographical region, age, and weight. The primary outcomes were pooled tibial nerve CSA in healthy controls, DM patients with and without DPN, and mean difference in the tibial nerve CSA across the three subgroups. The secondary outcomes included subgroup analysis by DPN criteria. A random-effects meta-analysis was applied. The effect sizes were calculated and reported with the 95% confidence intervals and standard deviation. Standard error values were calculated using the following equation: SE = SD/√(study size). Study heterogeneity was examined using I^2^-statistics. Within-subgroup differences were evaluated using *Q*-statistics. Publication bias was evaluated using a funnel plot and Egger’s regression test. Regression analysis was performed to study the influence of average age on mean tibial nerve CSA.

The meta-analysis and all calculations were performed using Stata version 17 (StataCorp). Statistical significance was established at *p =* 0.05 (two-tailed).

## 3. Results

### 3.1. Literature Search Results and Demography of the Subjects

The systematic literature search yielded a total of 1770 entries on Google Scholar, 309 entries on PubMed, and 545 entries on Scopus (Figure 1). A total of 130 duplicated entries from PubMed and Scopus were excluded. Initial abstract screening yielded a total of 129 potential studies, and the full-text of those studies were obtained and thoroughly explored. There were 113 that were further excluded after the full-texts were explored in detail including 106 unrelated studies, 2 case reports, and 5 studies in which the standard deviation was not reported. Finally, there were 16 studies eligible for meta-analysis of pooled tibial nerve CSA and the mean difference between DM and DPN patients.

In total, 16 studies [8,9,10,11,13,14,15,16,24,25,26,27,28,29,30,31] were included in the meta-analysis of pooled estimates and the mean difference of tibial nerve CSA (Table 1) (Table S1). Note that a single study may contain one to several groups of subjects categorized by diabetic status (DM or DPN) and age. Fourteen studies yielded a total of 3295 subjects in which 505 (15.3%) were healthy controls, 592 (18.0%) were diabetic patients without neuropathy, and 2198 (66.7%) were diabetic patients diagnosed with DPN. A total of 3125 subjects (94.9%) were diagnosed with type 2 diabetes. There were 50 subjects (1.5%) that were diagnosed with type 1 diabetes and 120 subjects (3.6%) diagnosed with either type 1 or type 2 diabetes. To diagnose the DPN, 8 diagnostic criteria (Table 1) were used including the American Diabetes Association Criteria (4 studies), Michigan Neuropathy Screening Instrument (3 studies), Toronto Clinical Neuropathy Score (3 studies), Total Neuropathy Score (1 study), Neuropathy Symptom Score and Neuropathy Disability Score (1 study), Diabetic Neuropathy Study Group in Japan (1 study), and Diabetic neuropathy score (1 study). Demographically, 2805 subjects were Asians, 80 were Europeans, 330 were North Americans, and 80 were Oceanians. Out of 16 studies, the study design of 15 studies was prospective, while there was only one retrospective study.

### 3.2. Quality Assessment

Quality assessment results including the proportions of studies with low, high, and unclear risk of bias are shown in Table 2. Regarding patient selection, 15 out of 16 studies fell into the low-risk category and only one study was in the high-risk category due to the retrospective study design. In terms of the index test and the reference standard, all 16 studies were classified as low risk because the diagnostic criteria of DPN was clearly stated. All studies were rated as low risk for the flow and timing category.

### 3.3. Tibial Nerve Cross-Sectional Area and Subgroup Analysis

**Figure 2:**
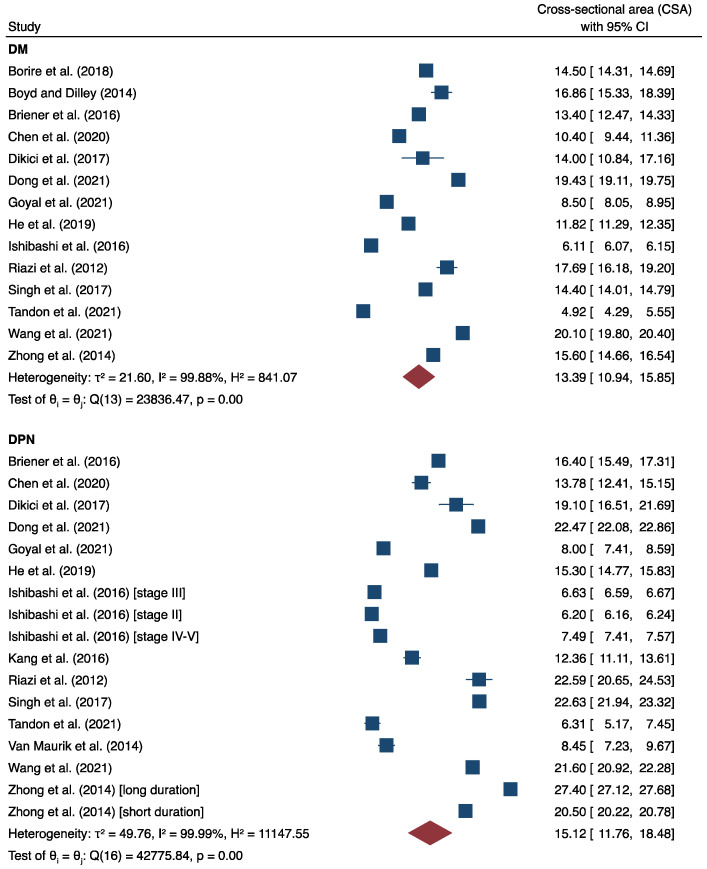

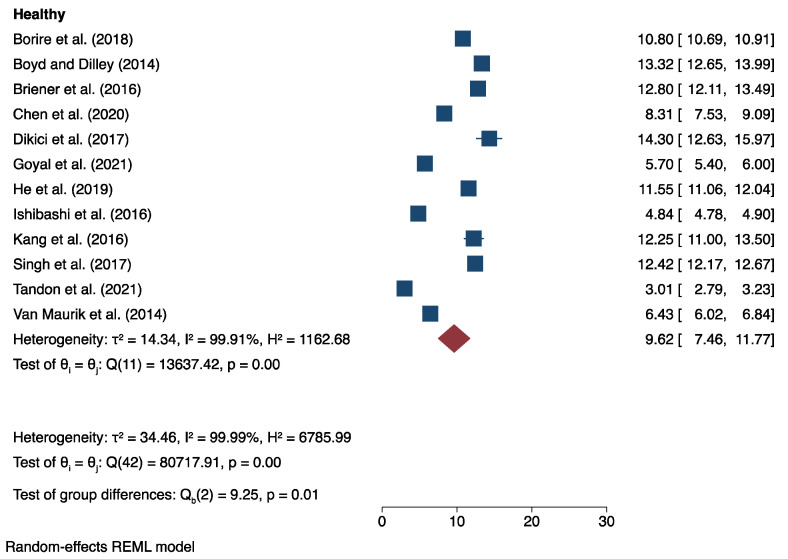
Forest plot showing the pooled tibial nerve cross-sectional area by subgroup. The squares indicate the cross-sectional areas from 16 studies [8,9,10,11,13,14,15,16,24,25,26,27,28,29,30,31]. Size of each square is relative to the study’s weight. Whiskers indicate the upper and lower limits. Red diamonds indicate the overall cross-sectional area in each subgroup.

**Figure 3:**
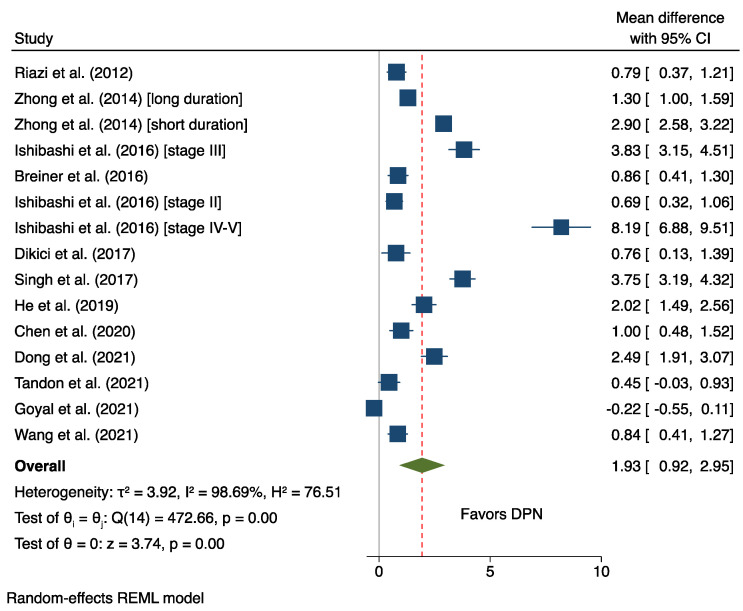
Mean differences comparing tibial nerve cross-sectional area between DPN and DM subgroups. The squares indicate the cross-sectional areas from 16 studies [8,9,10,11,13,14,15,16,24,25,26,27,28,29,30,31]. Size of each square is relative to the study’s weight. Whiskers indicate the upper and lower limits. The green diamond and dotted line indicate the overall cross-sectional area in each subgroup.

Pooled tibial nerve CSA is depicted in Figure 2. For DM patients, the mean tibial nerve CSA value was 13.39 mm^2^ (CI: 10.94–15.85, I^2^ = 99.88%). The mean tibial nerve CSA value for DPN was 15.12 mm^2^ (CI: 11.76–18.48, I^2^ = 99.99%). For healthy controls, the mean tibial nerve CSA value was 9.62 mm^2^ (CI: 7.76–11.77, I^2^ = 99.91%). Mean differences were calculated to compare the difference in the tibial nerve CSA of DM and DPN patients. We found that the tibial nerve CSA of DPN patients was 1.93 mm^2^ (CI: 0.92–2.95, I^2^ = 98.69%) larger than that of diabetic patients without DPN (*p* < 0.01) (Figure 3). Likewise, the tibial nerve CSA of DM patients was 2.16 mm^2^ (CI: 0.38–3.94, I^2^ = 99.16%) larger than healthy individuals (*p* < 0.01) (Figure S1). Finally, the tibial nerve CSA of DPN patients was approximately 4.32 mm^2^ (CI: 1.41–7.24, I^2^ = 99.61%) larger than healthy controls (Figure S1). Funnel plot pooled tibial nerve CSA was statistically symmetrical (Figure S2) (z = 1.79, *p* = 0.07), indicating no moderation by publication bias. Subgroup analysis showed that tibial nerve CSA varied considerably depending on the criteria used to diagnose DPN (Figure S3). The average tibial nerve CSA in the DPN patients diagnosed using the Simple Diagnostic Criteria proposed by the Diabetic Neuropathy Study Group in Japan and the Diabetic Neuropathy Score was only 6.77 mm^2^ (CI: 6.03–7.52, I^2^ = 99.89%) and 8.45 mm^2^ (CI: 7.23–9.67, I^2^ = 0.00%), respectively. On the other hand, the tibial nerve CSA of DPN patients diagnosed using other scoring systems was at least two- to three-times higher (Figure S3).

### 3.4. Correlational and Statistical Analyses

**Figure 4:**
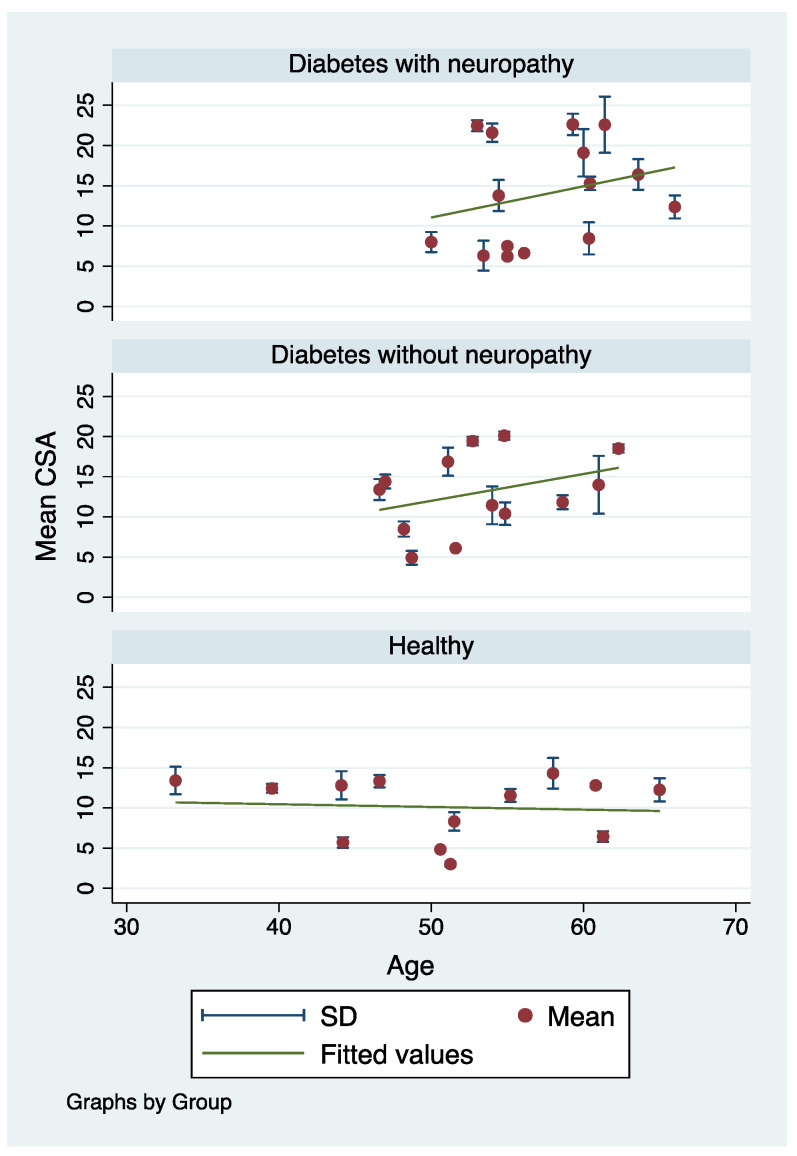
Correlation between mean tibial nerve CSA and age in diabetic patients, diabetic patients with neuropathy, and healthy controls.

Correlational analysis was performed to assess whether age or weight had any impact on tibial nerve CSA. We found that, although not statistically significant, there was a weakly positive trend between age and tibial nerve CSA for both diabetic patients (r = 0.35, *p* = 0.24) and diabetic patients with DPN (r = 0.27, *p* = 0.34), although it was not statistically significant (Figure 4). Correlation analysis between weight and tibial nerve CSA was not performed because of the limited number of studies available.

## 4. Discussion

An evidence-based synthesis of ultrasonographic tibial nerve CSA at the ankle is reported for the first time in this study. We found that tibial nerve CSA was 1.93 mm^2^ or 11% significantly larger in diabetic patients with DPN when compared with baseline DM patients (Figure 3). Nevertheless, there is still considerable overlap between the two groups (Figure 2), thus limiting the clinical usefulness of tibial CSA as a potential diagnostic marker of DPN at the moment. We believe that overlap might result from extreme between-study heterogeneity. Several reasons have been speculated as the underlying causes of heterogeneity. Most importantly, these criteria were not consistent among studies. Subgroup analysis by DPN criteria indicated that tibial nerve CSA was at least two-times lower in the DPN patients diagnosed using the Simple Diagnostic Criteria proposed by the Diabetic Neuropathy Study Group in Japan and the Diabetic Neuropathy Score than the other scoring systems (Figure S3).

In addition, tibial nerve CSA may be mediated by other factors including DPN severity, age, obesity, and status of diabetes. Previous studies showed that the increase of tibial nerve CSA was dependent on DPN stage [13,27] and duration [31]. A recent meta-analysis also found that symptoms of peripheral neuropathy may be apparent even before the diagnosis of diabetes [32], which may in part contribute to the marked heterogeneity of the results. Although the correlation between tibial nerve CSA and hemoglobin A1C or body mass index was not studied because these two parameters were reported by very few studies, a previous study found that the echo intensity of the sciatic nerve of diabetic rats was significantly increased at the fourth month of hyperglycemia, which was explained by the increase in water content, leading to nerve swelling [33]. Blood triglyceride levels in DM patients were also associated with DPN severity [34]. In addition, we cannot rule out the possibility of compression-related swelling rather than the systemic effect resulting from DM or DPN. It was found that diabetes is a risk factor for carpal tunnel syndrome, leading to median nerve enlargement [35], which may be similar to the posterior tibial nerve when entering the tarsal tunnel. In this study, we observed a positive association between age and tibial nerve CSA in the DM and DPN group (Figure 4), although the correlations were not statistically significant, plausibly due to the small sample sizes. Such an association may be related to DM or DPN duration rather than age itself. This explains why the increase in tibial nerve CSA was observed only in the DM and DPN groups, but not in the healthy controls (Figure 4). Cartwright, et al. [36] found significant correlation between tibial nerve CSA and weight. It was discussed in another study that heavier individuals generally had a greater amount of tissue overall, which presumably led to a higher amount of tissue within the nerves [37]. Furthermore, the standardized protocol for nerve evaluation in the lower extremities is currently lacking [38]. We observed that the location used to carry out the ultrasound measurement also varied considerably, ranging from 1 to 7 cm proximal to the medial malleolus. These data indicated that changes to the tibial nerve CSA are multicausal and may be moderated by a several factors, leading to the high heterogeneity of the results. Therefore, the clinical application of tibial nerve CSA for the diagnosis of DPN may be limited.

The present study is not without limitations. Most of the patients included in the present study were of Asian origin, making the results heavily biased towards a single group of population, which may not be generalizable to the global population. Furthermore, no children with diabetes or DPN were included in the present meta-analysis. To bridge the age gap, sonographic measurements in young diabetic patients may be worth for future exploration. Due to the high anatomical variability of the tibial nerve at the popliteal fossa or above, pooled estimates of tibial nerve CSA at different locations were not analyzed. The results of the present meta-analysis were associated with high between-study heterogeneity, and the results should be interpreted with caution. While high-resolution ultrasound may offer a more convenient alternative to nerve conduction studies to diagnose DPN, it may not be available in many primary care settings

## 5. Conclusions

In summary, the analysis of 3018 individuals from 16 studies indicated that tibial nerve CSA at the ankle is significantly larger in DPN patients when compared with diabetic patients without DPN. However, our data indicated that it may not be sensitive enough due to considerable overlap between the two groups as tibial nerve CSA could further be mediated by other factors such as age, diabetic status, obesity, DM duration, DPN severity, and the criteria used to diagnose DPN. The present study also reiterates the need for a standardized protocol for the evaluation of nerves in the lower extremities, as well as consistency in the criteria used to diagnose DPN.

## Figures and Tables

**Table 1:** Characteristics of the 16 included studies for the meta-analysis of pooled effect size and mean difference of tibial nerve cross-sectional area in diabetic patients with diabetic neuropathy, diabetic patients, and healthy controls. Studies are listed in alphabetical order.

Author	Year	Country	Criteria Used to Diagnose DPN	Group	Participants’ Characteristics		
					Number	Age (yr)	Weight (kg)
Borire et al. [29]	2018	Australia	Total Neuropathy Score	Healthy	60	62.3	NR
				Diabetes	30	60.8	NR
Boyd and Dilley [24]	2014	USA	Michigan Neuropathy Screening Instrument and Michigan Diabetic Neuropathy Score	Healthy	20	46.6	72.59
				Diabetes	20	51.1	84.87
Breiner et al. [8]	2016	USA	American Diabetes Association criteria	Healthy	100	44.1	NR
				Diabetes	30	46.6	NR
				DPN	67	63.3	NR
Chen et al. [25]	2020	China	American Academy of Electrodiagnostic Medicine	Diabetes	33	54.85	NR
				DPN	30	54.43	NR
				Healthy	33	51.51	NR
Dikici et al. [9]	2017	Turkey	American Diabetes Association criteria	Diabetes	20	61	NR
				DPN	20	60	NR
				Healthy	20	58	NR
Dong et al. [30]	2021	China	Michigan Neuropathy Screening Instrument	Diabetes	38	52.72	NR
				DPN	42	53.02	NR
Goyal et al. [26]	2021	India	Toronto Clinical Neuropathy Score	Healthy	70	44.2	66.9
				Diabetes	70	48.2	70.2
				DPN	70	50.3	68.1
He et al. [10]	2019	China	Neuropathy Symptom Score and Neuropathy Disability Score	Diabetes	40	58.63	61.21
				DPN	40	60.43	62.98
				Healthy	40	55.2	55.83
Ishibashi et al. [27] ^a^	2016	Japan	Simple Diagnostic Criteria by Diabetic Neuropathy Study Group in Japan	Diabetes	50	51.6	NR
				DPN (stage II)	71	55	NR
				DPN (stage III)	43	56.1	NR
				DPN (stages IV-V)	34	55	NR
				Healthy	29	50.6	NR
Kang et al. [11]	2016	South Korea	American Academy of Electrodiagnostic Medicine	DPN	20	66	59.87
				Healthy	20	65	60.25
Riazi et al. [13]	2012	USA	Toronto Clinical Neuropathy Score	Diabetes	43	46.8	NR
				DPN	50	61.4	NR
Singh et al. [14]	2017	India	Toronto Clinical Neuropathy Score	Diabetes	75	46.98	63.24
				DPN	58	59.3	78.52
				Healthy	75	39.54	65.34
Tandon et al. [15]	2020	India	American Diabetes Association criteria	Diabetes	29	48.72	62.18
				DPN	41	53.43	66.95
				Healthy	30	51.26	61.5
Van Maurik et al. [16]	2014	Netherlands	Diabetic Neuropathy Score	DPN	42	60.36	89.76
				Healthy	38	61.29	70.84
Wang et al. [28]	2021	China	Michigan Neuropathy Screening Instrument	Diabetes	44	54.77	NR
				DPN	44	54.05	NR
Zhong et al. [31] ^a^	2014	China	American Diabetes Association criteria	Diabetes	50	NR	NR
				DPN (short duration)	715	NR	NR
				DPN (long duration)	811	NR	NR

^a^ In these studies, subjects were divided into subgroups according to the stage or duration of diabetic neuropathy. NR: not reported, DPN: diabetic peripheral neuropathy, USA: United States of America.

**Table 2:** Risk assessment using QUADAS-2 tool.

Study	Patient Selection	Index Test	Reference Standard	Flow and Timing
Borire et al. [29]	Low	Low	Low	Low
Boyd and Dilley [24]	Low	Low	Low	Low
Breiner et al. [8]	Low	Low	Low	Low
Chen et al. [25]	Low	Low	Low	Low
Dikici et al. [9]	Low	Low	Low	Low
Dong et al. [30]	Low	Low	Low	Low
Goyal et al. [26]	Low	Low	Low	Low
He et al. [10]	Low	Low	Low	Low
Ishibashi et al. [27]	Low	Low	Low	Low
Kang et al. [11]	Low	Low	Low	Low
Riazi et al. [13]	Low	Low	Low	Low
Singh et al. [14]	Low	Low	Low	Low
Tandon et al. [15]	Low	Low	Low	Low
Van Maurik et al. [16]	Low	Low	Low	Low
Wang et al. [28]	High	Low	Low	Low
Zhong et al. [31]	Low	Low	Low	Low

## Data Availability

The data that support the findings of this study are available from the corresponding author upon reasonable request.

## Supplementary Materials

The following Supporting Information can be downloaded at: https://www.mdpi.com/1648-9144/58/12/1696/s1, Table S1: List of studies included in the meta-analysis of tibial nerve CSA in healthy subjects, Figure S1: Forest plots of mean difference between DM group vs. DPN group, healthy group vs. DM group, and healthy group vs. DPN group, Figure S2: Funnel plots of pooled tibial nerve CSA and mean difference, Figure S3: Subgroup analysis of tibial nerve CSA by diagnostic criteria.
