# Supplementary figures and images for: Cross-Sectional Area of the Tibial Nerve in Diabetic Peripheral Neuropathy Patients: A Systematic Review and Meta-Analysis of Ultrasonography Studies
Source: Medicina (Kaunas). 2022 Nov 22;58(12):1696. doi: 10.3390/medicina58121696 (PMC9787041; doi:10.3390/medicina58121696)

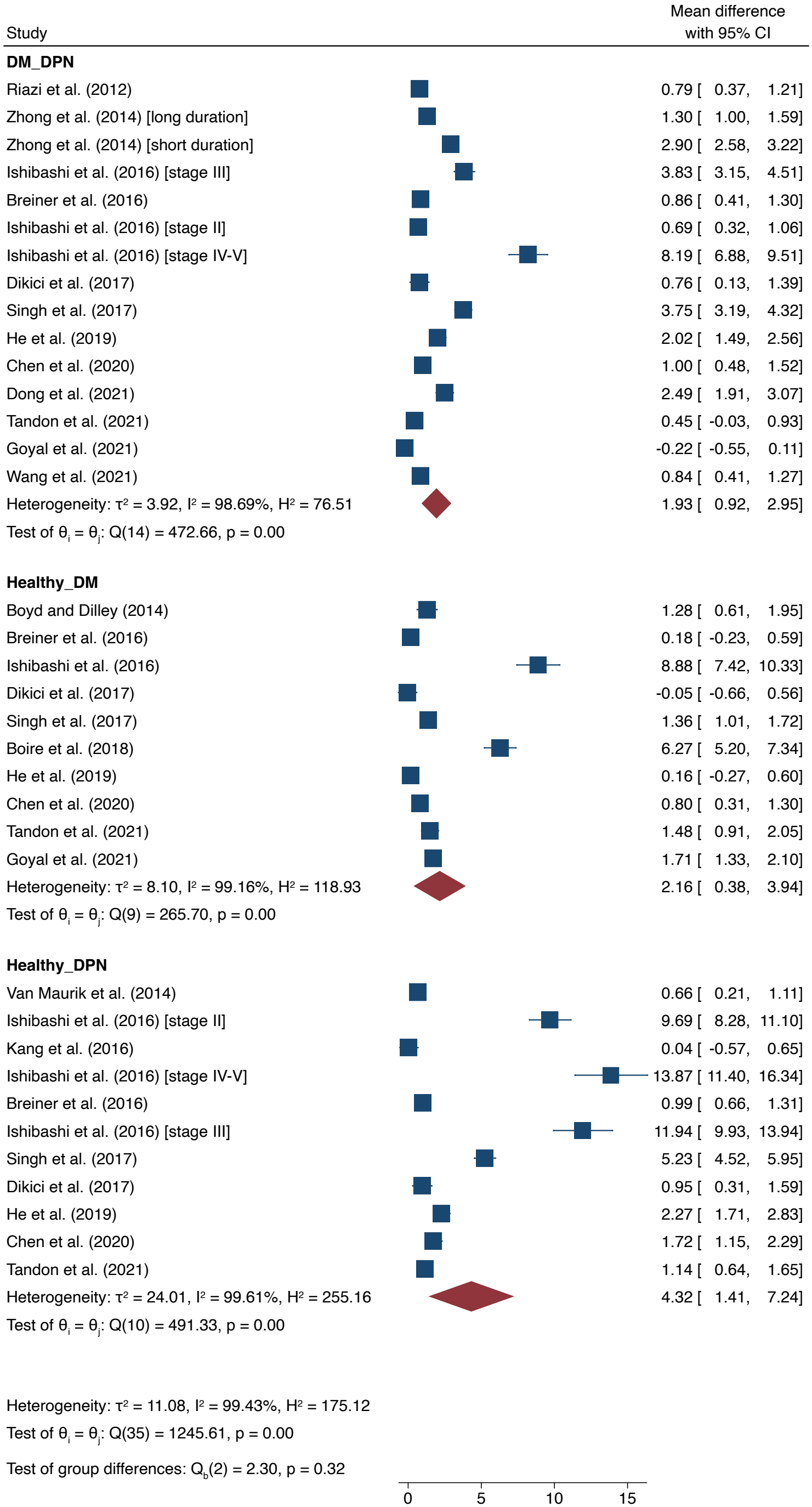

Supplement: Supplementary file 1 [file medicina-58-01696-s001.zip › Fig S1.pdf]

Funnel plot

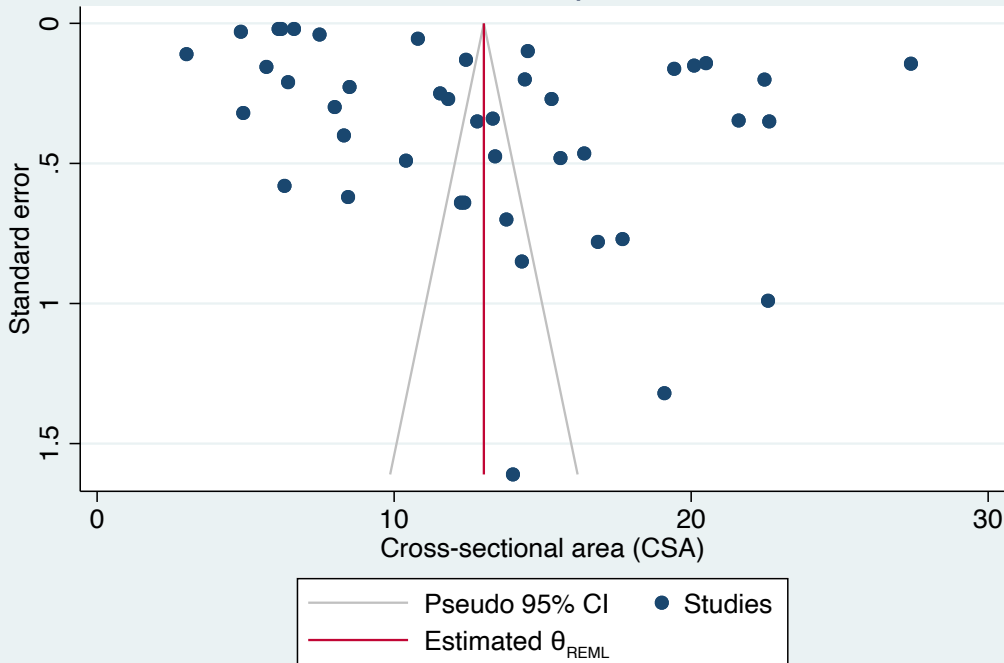

Supplement: Supplementary file 1 [file medicina-58-01696-s001.zip › Fig S2.pdf]

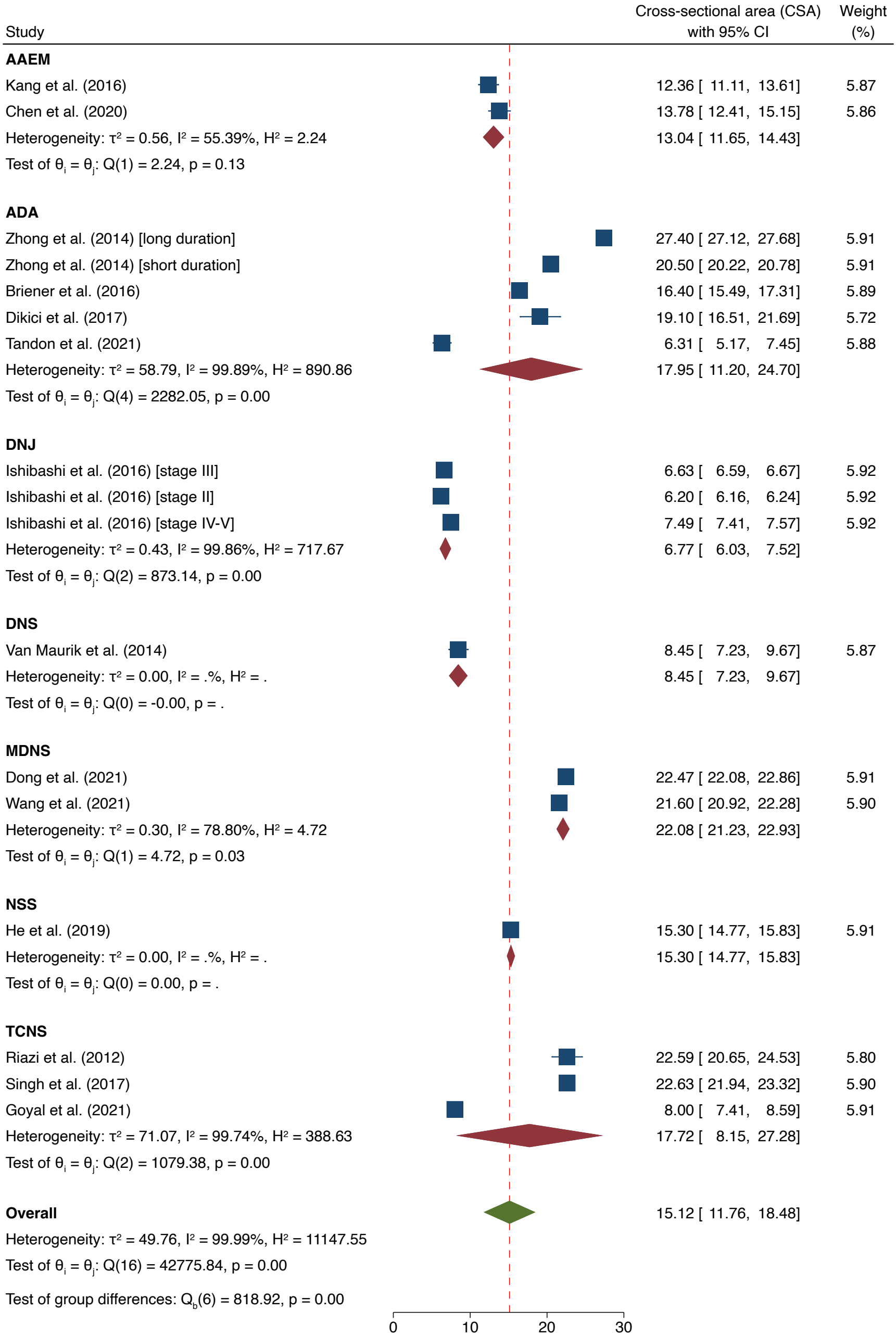

Supplement: Supplementary file 1 [file medicina-58-01696-s001.zip › Fig S3.pdf]
